# Association between medical androgen deprivation therapy and long‐term cardiovascular disease and all‐cause mortality in nonmetastatic prostate cancer

**DOI:** 10.1002/ijc.34058

**Published:** 2022-05-17

**Authors:** Rachel B. Forster, Anders Engeland, Rune Kvåle, Vidar Hjellvik, Tone Bjørge

**Affiliations:** ^1^ Department of Health Registry Research and Development Norwegian Institute of Public Health Bergen Norway; ^2^ Department of Chronic diseases Norwegian Institute of Public Health Oslo Norway; ^3^ Department of Global Public Health and Primary Care University of Bergen Bergen Norway; ^4^ Department of Oncology and Medical Physics Haukeland University Hospital Bergen Norway; ^5^ Cancer Registry of Norway Oslo Norway

**Keywords:** androgen deprivation therapy, cardiovascular disease, epidemiology, prostate cancer

## Abstract

Studies have suggested that prostate cancer (PCa) patients receiving androgen deprivation therapy (ADT) are at increased risk of developing or exacerbating cardiovascular disease (CVD). We aimed to explore the association between ADT for PCa and subsequent CVD and all‐cause mortality in this nationwide, longitudinal study. We also evaluated the role of cardiovascular risk and ADT duration to determine effect modification. Norwegian registry data were used to identify patients with PCa from 2008‐18 and who received primary ADT in the first year after diagnosis. The associations between ADT and composite cardiovascular events, and the individual components of myocardial infarction, stroke and heart failure, in addition to atrial fibrillation and all‐cause mortality, were explored using time‐varying Cox regression models. We included 30 923 PCa patients, of whom 8449 (27%) received primary ADT. Mean follow‐up was 2.9 and 3.8 years for CVD events and mortality, respectively. We found an association between ADT and composite CVD (adjusted HR 1.13: 95% CI 1.05‐1.21), myocardial infarction (1.18: 1.05‐1.32), stroke (1.21: 1.06‐1.38), heart failure (1.23: 1.13‐1.35) and all‐cause mortality (1.49: 1.39‐1.61). These associations persisted in those with low and moderate CVD risk and ADT longer than 7 months. A relationship between ADT and composite CVD and all‐cause mortality was observed, especially in those with moderate CVD risk and longer treatment duration. Future studies with more detailed cancer data are needed to verify the clinical relevance of these results, especially when considering all‐cause mortality within the context of treatment guidelines and benefits of ADT.

## Graphical abstract

What's new?

Existing evidence suggests that prostate cancer patients receiving androgen deprivation therapy are at increased risk of cardiovascular disease. The relationship between prostate cancer treatment and cardiovascular disease remains unclear, however. Using longitudinal registry data for all prostate cancer patients in Norway, our study identifies an increased risk of cardiovascular disease and mortality in patients treated with androgen deprivation therapy. The increased risk is most pronounced in patients with low or moderate prior risk of cardiovascular disease and longer duration of treatment. The findings contribute new evidence to the ongoing discussion about the use of hormonal therapy.

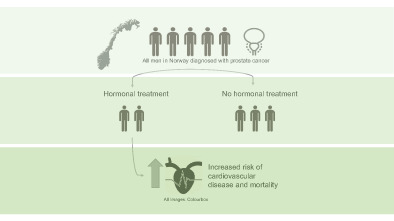

**Abbreviations**
ADT: androgen deprivation therapy
CI: confidence interval
CVD: cardiovascular disease
EAU: European Association of Urology
HR: hazard ratio
LHRH: leutenizing hormone‐releasing hormone
NorPD: Norwegian Prescription Database
NTproBNP: N‐terminal pro‐B‐type natriuretic peptide
PCa: prostate cancer

## 1 INTRODUCTION

Prostate cancer (PCa) patients have increased risk of developing cardiovascular disease (CVD) and are more likely to die from non‐PCa specific causes, mainly cardiovascular related, than from PCa^1^, ^2^ There is evidence that androgen deprivation therapy (ADT), a common treatment for more aggressive PCa, may increase the risk of cardiovascular morbidity and mortality, as well as all‐cause mortality.^2^, ^3^, ^4^, ^5^, ^6^ However, there have been contradictory findings of the relationship between PCa treatment and CVD.^7^, ^8^, ^9^, ^10^

Contradictory findings come mainly from randomised controlled trials which historically include healthier patients than the general population and may not reflect real‐world experiences.^11^ The hypothesis that increased risk of CVD after ADT disproportionately affects those with preexisting CVD is reflected in studies that have shown increased risk of all‐cause mortality after treatment with ADT only in those with a higher burden of CVD comorbidity.^12^, ^13^ Also, studies showing no association between ADT and adverse health outcomes have mainly focused on all‐cause mortality or fatal CVD rather than nonfatal CVD events. Thus, they cannot directly provide evidence on whether ADT increases the risk of nonfatal CVD.^7^, ^8^, ^9^

ADT has been a cornerstone of treatment for PCa for decades, with an aim to deprive androgen‐dependent prostate cancer cells of circulating androgen and its precursors.^14^ This treatment is currently recommended as monotherapy or in combination with other therapeutics for aggressive and more advanced cancers and has shown to be greatly beneficial at reducing cancer related death, but also the cause of some significant side effects.^15^, ^16^ It is therefore imperative to determine if this critical treatment option is contributing to increased risk of CVD, especially in those with higher risk of CVD, and determine if there are appropriate treatment recommendation modifications that should be made to mitigate this risk.

The aim of our study was to use longitudinal Norwegian health registry data that reflects real‐life application in a nationwide cohort of PCa patients to explore the association between primary medication‐based ADT for PCa and subsequent CVD and all‐cause mortality. We also aimed to evaluate the role of cardiovascular risk prior to PCa to determine effect modification by CVD risk, as well as ADT duration.

## 2 METHODS

### 2.1 Study population

This is a registry‐based, longitudinal cohort study using nationwide data from Norway (population 2.0 million males above age 20 years in 2018). A unique national ID number for all residents in Norway allowed for comprehensive, encrypted linkage between the registries.

Data on PCa diagnosis were extracted from the Cancer Registry of Norway (1953‐2018, data from 1953 to 2007 was used to determine prior cancers), reimbursed prescription data was sourced from the Norwegian Prescription Database (NorPD) (2004‐2019, with diagnosis codes included from 2008), the Norwegian Patient Registry provided specialist health care data (2008‐2018), the National Population Registry provided data on emigration and deaths (2005‐2019), and the Norwegian National Education Database provided information on highest education achieved (2004, 2009 and 2014).

The cohort included all males born during 1924‐1990, alive and living in Norway on 1 January 2009, and with PCa diagnosed 2008‐2018 and no other cancer diagnosis prior to PCa. For this analysis, we have restricted our population to PCa diagnosis years 2008‐2017 (CVD outcomes) or 2008‐2018 (mortality). We relied on the Norwegian Patient Registry and NorPD to derive risk factors and CVD outcomes, which holds individual level data from 2008. This is also the year the prescription data began including reimbursement codes corresponding to diagnoses.

Patients were excluded if they had a cancer morphology not consistent with malignant adenocarcinomas of the prostate, evidence of distant metastases at diagnosis or unknown stage, if they had less than 1 year of follow‐up, if they received primary treatment (within the first year of diagnosis) with abiraterone or enzalutamide, or any ADT prior to their PCa diagnosis.

The ADT group included patients that received primary treatment with a leutenizing hormone‐releasing hormone (LHRH) agonist (buserelin, leuprorelin, goserelin, triptorelin or histrelin) or LHRH antagonists (degarelix or abarelix) within the first year after diagnosis. The treatment group was compared to PCa patients that did not receive ADT within the first year.

### 2.2 Follow‐up and outcomes

Follow‐up began 1 year after PCa diagnosis and continued until one of the following: a CVD outcome (listed below), death, emigration, aged over 85 years or end of study on 31 December 2018 for CVD outcomes and 31 December 2019 for all‐cause mortality. If the specific CVD outcome occurred in the year between PCa diagnosis and when the follow‐up started, that patient was excluded from the analysis.

Cardiovascular and mortality outcomes included (full definitions using diagnostics codes are provided in Supplemental A):

1. Composite CVD (myocardial infarction, stroke and/or heart failure).
2. Myocardial infarction.
3. Stroke.
4. Heart failure.
5. Atrial fibrillation.
6. All‐cause mortality.

### 2.3 Covariates

Other variables we included in our statistical models were age (continuous) at PCa diagnosis, cancer stage (local or regional), highest education level completed at diagnosis (compulsory, intermediate, tertiary), year of PCa diagnosis (2008‐2010, 2011‐2013, 2014‐2017/2018) and the presence of CVD risk factors in the year prior to PCa diagnosis: Hypertension, hypercholesterolemia, atrial fibrillation, heart failure, myocardial infarction, stroke or diabetes. Further information on the definition of the CVD risk factor variables can be found in Supplemental A.

We also created a prescription‐based comorbidity index using prescription data from the 4 years prior to PCa diagnosis (Supplemental B), based on methods developed by Häppölä et al., to account for multimorbidity.^17^ The original study found the score to perform similarly to the Charlson comorbidity index, but not be reliant on extensive clinical information. We validated the derived index with all‐cause mortality and found a consistent, positive association. The comorbidity index was created as a continuous variable that ranged from −4 to 9 in our population but then used in analysis as a categorical variable: <0, 0, 1‐4 or ≥5.

### 2.4 Statistical analysis

Cox proportional hazards regression models with time since diagnosis as a time‐variable were used to estimate the association between ADT and the CVD outcomes and all‐cause mortality, reporting hazard ratios (HRs) and 95% confidence intervals (CIs).

The fully adjusted multivariable models included ADT status (treated/not treated), age at PCa diagnosis, stage, year of PCa diagnosis, education, comorbidity index and CVD risk factors in the year prior to PCa diagnosis: hypertension, hypercholesterolemia, diabetes, atrial fibrillation, heart failure, myocardial infarction and stroke.

Next, we wanted to explore the impact of CVD risk at the time of PCa diagnosis more thoroughly. A statistical model with covariates corresponding to the fully adjusted model above, but without the CVD risk factors included was constructed, and was stratified by the CVD risk groups based on risk factors present in the year prior to PCa diagnosis:

- Low risk:
  - No evidence of prior CVD event (myocardial infarction, stroke, heart failure), no hypertension, no hypercholesterolemia, no diabetes.
- Medium risk:
  - Hypertension.
  - AND/OR hypercholesterolemia.
  - AND/OR diabetes.
  - AND NO stroke or myocardial infarction or heart failure.
- High risk:
  - Prior stroke.
  - AND/OR myocardial infarction.
  - AND/OR heart failure.

We also evaluated the impact of duration of ADT by including the ADT variable as <7 months, 7‐18 months and >18 months compared to no use, as a time‐varying covariate. Duration of ADT was determined by prescription patterns, where duration of continuous prescriptions was added together. If there was longer than 6 months between one prescription and the following the treatment period was ended. Six months is the longest duration possible on a single prescription for most of the various treatment options in our study (Supplemental C).

A final set of models were conducted incorporating CVD risk and ADT duration groups. Sensitivity analysis was performed by removing people diagnosed with PCa in 2008 to make sure possible missing CVD risk factor data in this group did not affect the outcomes. Additional post hoc sensitivity analysis was performed on the models stratified by CVD risk by removing patients in the ADT treatment group that received LHRH antagonist medications. All analyses were performed using SPSS (version 27) and STATA (version 15.0) software.

## 3 RESULTS

**FIGURE 1:**
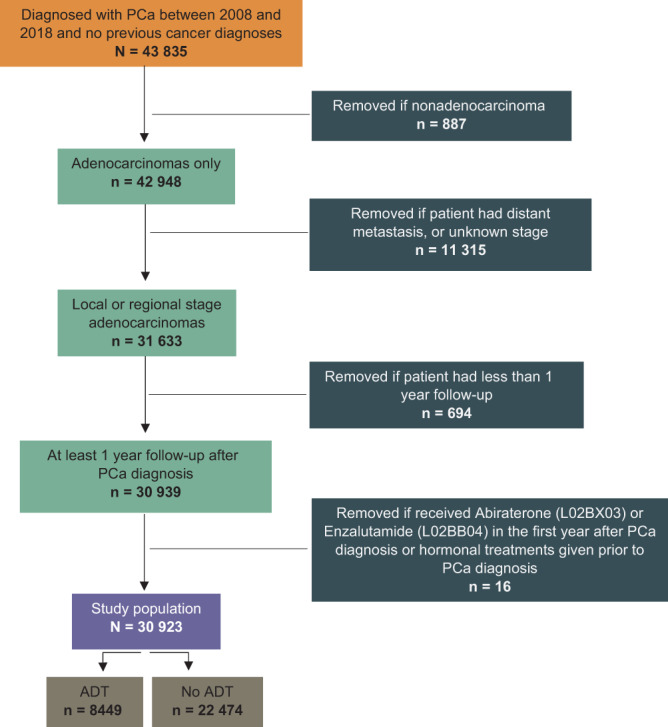
Selection of the study population (including cancer‐free males living in Norway January 1st, 2008) [Color figure can be viewed at wileyonlinelibrary.com]

A total of 43 835 males born during 1924‐1990, aged 37‐84, were diagnosed with PCa during 2008‐2018 with no prior cancer diagnoses, and after applying the exclusion criteria 30 923 were included in the cohort (Figure 1). Of these, 8449 received ADT in the first year after PCa diagnosis.

From Table 1, the mean age of the whole study population was 67.4 years, while those that received ADT were slightly older at 70.8 years than those that did not receive primary ADT (66.1 years). Most patients that received ADT had regional disease where most that did not had localised disease. Highest educational level achieved was roughly similar between the two groups while there appeared to be higher proportions of comorbidity in the ADT group, which was also demonstrated in the proportion of CVD risk factors.

**TABLE 1:** Study characteristics at time of prostate cancer diagnosis

	Total (n = 30 923)	ADT (n = 8449)	No ADT (n = 22 474)
Mean age at diagnosis (years [SD])	67.4 (7.6)	70.8 (6.8)	66.1 (7.5)
Age at diagnosis (years)			
<60	5243 (17.0%)	563 (6.7%)	4680 (20.8%)
60‐64	6260 (20.2%)	1122 (13.3%)	5138 (22.9%)
65‐69	8130 (26.3%)	1962 (23.2%)	6168 (27.4%)
70‐84	11 290 (36.5%)	4802 (56.8%)	6488 (28.9%)
Stage			
Local	19 983 (64.4%)	3595 (42.5%)	16 388 (72.9%)
Regional	10 940 (35.4%)	4854 (57.5%)	6086 (27.1%)
Education level^a^			
Compulsory	6659 (21.5%)	2262 (26.8%)	4397 (19.6%)
Intermediate	15 825 (51.2%)	4326 (51.2%)	11 499 (51.2%)
Tertiary	8319 (26.9%)	1820 (21.5%)	6499 (28.9%)
Unknown	120 (0.4%)	41 (0.5%)	79 (0.4%)
Comorbidity score			
<0	3954 (12.8%)	949 (11.2%)	3005 (13.4%)
0	16 438 (53.2%)	3976 (47.1%)	12 462 (55.5%)
1‐4	10 274 (33.2%)	3412 (40.4%)	6862 (30.5%)
5+	257 (0.8%)	112 (1.3%)	145 (0.6%)
Year of prostate cancer diagnosis			
2008‐2010	7145 (23.1%)	2255 (26.7%)	4890 (21.8%)
2011‐2013	9485 (30.7%)	2723 (32.2%)	6762 (30.1%)
2014‐2018	14 293 (46.2%)	3471 (41.1%)	10 822 (48.2%)
Hypertension (yes)	15 710 (50.8%)	4891 (57.9%)	10 819 (48.1%)
High cholesterol (yes)	10 566 (34.2%)	3348 (39.6%)	7218 (32.1%)
Diabetes	2922 (9.4%)	1009 (11.9%)	1913 (8.5%)
Heart failure	1640 (5.3%)	615 (7.3%)	1025 (4.6%)
Atrial fibrillation	2553 (8.3%)	938 (11.1%)	1615 (7.2%)
Myocardial infarction	1210 (3.9%)	415 (4.9%)	795 (3.5%)
Stroke	853 (2.8%)	343 (4.1%)	510 (2.3%)
CVD risk			
Low	12 508 (40.4%)	2841 (33.6%)	9667 (43.0%)
Medium	15 242 (49.3%)	4433 (52.5%)	10 809 (48.1%)
High	3173 (10.3%)	1175 (13.9%)	1998 (8.9%)

Abbreviations: ADT, androgen deprivation therapy; CVD, cardiovascular disease.

^a^ Categorisation based on information from Statistics Norway; information from 2004 was used for men diagnosed in 2008, information from 2009 was used for men diagnosed in 2009‐2013 and information from 2014 was used for men diagnosed in 2014‐2018.

By the end of the study 4445 (14%) experienced a first CVD outcome during follow‐up (2009‐2017), 3360 (11%) died (2009‐2018), 53 (0.2%) had emigrated and 2103 (6.8%) were censored at 85 years. The mean follow‐up was 2.9 years (range 0‐10) for those that experienced a CVD outcome during follow‐up and 3.8 years (range 0‐11) for those that died.

For the patients that received ADT in the first year after PCa diagnosis, 1460 (17%) had less than 7 months of hormonal treatment, 4755 (56%) had 7‐18 months and 2234 (26%) had more than 18 months.

### 3.1 Association between ADT and CVD and death by CVD risk groups

In the fully adjusted model, there was an association between ADT and increased risk of composite CVD with an HR of 1.13 (95% CI 1.05‐1.21), and this association was maintained in both the low and moderate CVD risk groups, and in the high CVD risk group a stronger association was indicated but not statistically significant (Table 2). There was also an association between ADT and risk of each of the component CVD outcomes that made up the composite CVD in the fully adjusted model: Myocardial infarction HR 1.18 (1.05‐1.32), stroke HR 1.21 (1.06‐1.38) and heart failure HR 1.23 (1.13‐1.35). These associations varied based on CVD risk groupings. There was no association with myocardial infarction in the low and high‐risk groups, but there was in the moderate CVD risk group (HR 1.17, 1.01‐1.36). There was also no association with stroke in the low and moderate‐risk groups, but there was an association in the high‐risk group: HR 1.42 (1.01‐2.00). Hormonal treatment and heart failure were associated across all three CVD risk groups: HR 1.30 (1.06‐1.58), 1.23 (1.10‐1.37) and 1.38 (1.04‐1.84), respectively.

**TABLE 2:** Risk of cardiovascular disease and death after hormonal treatment for prostate cancer

Outcome	Age‐adjusted HR (95% CI)	Full multivariable^a^ HR (95% CI)	Low CVD risk^b^ HR (95% CI)	Moderate CVD risk^b^ HR (95% CI)	High CVD risk^b^ HR (95% CI)
Composite CVD	1.17 (1.09‐1.25)	1.13 (1.05‐1.21)	1.17 (1.03‐1.33)	1.11 (1.02‐1.21)	1.29 (0.92‐1.82)
Myocardial infarction	1.23 (1.10‐1.38)	1.18 (1.05‐1.32)	1.24 (0.97‐1.57)	1.17 (1.01‐1.36)	1.13 (0.83‐1.54)
Stroke	1.23 (1.08‐1.40)	1.21 (1.06‐1.38)	1.23 (0.96‐1.58)	1.13 (0.95‐1.35)	1.42 (1.01‐2.00)
Heart failure	1.36 (1.24‐1.48)	1.23 (1.13‐1.35)	1.30 (1.06‐1.58)	1.23 (1.10‐1.37)	1.38 (1.04‐1.84)
Atrial fibrillation	1.06 (0.97‐1.16)	1.03 (0.94‐1.13)	0.96 (0.80‐1.14)	1.04 (0.92‐1.17)	1.10 (0.85‐1.42)
All‐cause mortality	1.78 (1.65‐1.91)	1.49 (1.38‐1.61)	1.75 (1.52‐2.01)	1.50 (1.35‐1.66)	1.17 (0.98‐1.39)

Abbreviations: CI, confidence interval; CVD, cardiovascular disease; HR, hazard ratio; PCa, prostate cancer.

^a^ Covariates in model (whole population): Treatment status (hormonal treatment/no hormonal treatment) + age at PCa diagnosis, period of diagnosis, cancer stage, education, comorbidity index, hypertension, high cholesterol, diabetes, atrial fibrillation, heart failure, myocardial infarction, stroke.

^b^ Covariates in model (stratified by CVD risk): Treatment status (hormonal treatment/no hormonal treatment) + age at PCa diagnosis, period of diagnosis, cancer stage, education, comorbidity index (CVD risk factors used to define risk groups).

In addition, all‐cause mortality was significantly increased in the ADT group with a HR of 1.49 (1.38‐1.61) in the fully adjusted multivariable model, as well as for each individual CVD risk group. There was no association between ADT and atrial fibrillation during the study period (HR 1.03: 0.94‐1.13).

### 3.2 Association between ADT and CVD and death, accounting for ADT duration

In the fully adjusted models, there were no associations between ADT <7 months and composite CVD or the individual events. However, there were associations between ADT lasting 7‐18 months and composite CVD, stroke and heart failure: HR 1.12 (1.03‐1.22), 1.32 (1.14‐1.54) and 1.25 (1.13‐1.39), respectively (Table 3). There was also an increased risk in composite CVD, myocardial infarction and stroke in the ADT group that received treatment over 18 months (HR 1.23 [1.09‐1.38], 1.31 [1.08‐1.60] and 1.28 [1.09‐1.49], respectively). There was also an association between >18 months treatment and atrial fibrillation, HR 1.18 (1.01‐1.37).

**TABLE 3:** Risk of cardiovascular disease and death after hormonal treatment for prostate cancer by duration of hormonal treatment

Outcome	ADT <7 months HR (95% CI)	ADT 7‐18 months HR (95% CI)	ADT >18 months HR (95% CI)
Composite CVD	1.07 (0.93‐1.23)	1.12 (1.03‐1.22)	1.23 (1.09‐1.38)
Myocardial infarction	1.10 (0.88‐1.38)	1.14 (0.99‐1.31)	1.31 (1.08‐1.60)
Stroke	1.09 (0.85‐1.41)	1.32 (1.14‐1.54)	1.03 (0.81‐1.30)
Heart failure	1.14 (0.96‐1.36)	1.25 (1.13‐1.39)	1.28 (1.09‐1.49)
Atrial fibrillation	1.00 (0.83‐1.20)	1.00 (0.90‐1.12)	1.18 (1.01‐1.37)
All‐cause mortality	1.52 (1.34‐1.74)	1.35 (1.23‐1.47)	2.04 (1.83‐2.27)

*Note*: Model includes age, period of diagnosis, cancer stage, education, comorbidity index and CVD risk factors (hypertension, high cholesterol, diabetes, atrial fibrillation, heart failure, myocardial infarction, stroke).

Abbreviations: ADT, androgen deprivation treatment (hormonal treatment); CI, confidence interval; CVD, cardiovascular disease; HR, hazard ratio.

All three treatment durations were associated with all‐cause mortality: <7 months HR 1.52 (1.34‐1.74), 7‐18 months HR 1.35 (1.23‐1.47) and >18 months HR 2.04 (1.83‐2.27).

### 3.3 Association between ADT (accounting for duration) and CVD and death, stratified by CVD risk at PCa diagnosis

When we further stratified our analysis by CVD risk at time of PCa diagnosis as either low‐, moderate‐ or high‐risk and ADT duration, we found an association with composite CVD for both low‐ and moderate‐risk CVD only in the >18 months ADT group (Table 4). Risk of myocardial infarction was also increased in the low CVD risk group in those that received ADT more than 18 months. Increased risk of heart failure was observed in the low‐risk group for <7 months and >18 months treatment duration, as well as in the moderate risk group for 7‐18 and >18 months, and only in the 7‐18 months duration group for high CVD risk. There remained an association for all durations of ADT and risk CVD groups with all‐cause mortality except for 7‐18 months duration in the high CVD risk group.

**TABLE 4:** Risk of cardiovascular disease and death after hormonal treatment for prostate cancer stratified by duration of hormonal treatment and cardiovascular risk at prostate cancer diagnosis

	Low CVD risk HR (95% CI)			Moderate CVD risk HR (95% CI)			High CVD risk HR (95% CI)		
Outcome	<7 months	7‐18 months	>18 months	<7 months	7‐18 months	>18 months	<7 months	7‐18 months	>18 months
Composite CVD	1.08 (0.85‐1.39)	1.16 (0.99‐1.35)	1.38 (1.13‐1.68)	1.02 (0.86‐1.20)	1.09 (0.98‐1.21)	1.17 (1.01‐1.36)	1.41 (0.79‐2.52)	1.25 (0.88‐1.79)	0.60 (0.29‐1.65)
Myocardial infarction	1.18 (0.74‐1.89)	1.14 (0.84‐1.54)	1.53 (1.06‐2.21)	1.03 (0.76‐1.39)	1.16 (0.97‐1.39)	1.27 (0.98‐1.64)	1.26 (0.74‐2.16)	1.10 (0.78‐1.55)	0.98 (0.56‐1.74)
Stroke	0.94 (0.56‐1.59)	1.43 (1.07‐1.92)	1.06 (0.69‐1.62)	1.22 (0.89‐1.67)	1.21 (0.99‐1.48)	0.85 (0.61‐1.18)	0.91(0.42‐1.96)	1.49 (1.02‐2.18)	1.64 (0.91‐2.97)
Heart failure	1.60 (1.14‐1.24)	1.19 (0.93‐1.52)	1.40 (1.03‐1.89)	1.06 (0.86‐1.32)	1.29 (1.14‐1.46)	1.24 (1.03‐1.49)	1.11 (0.60‐2.05)	1.37 (1.00‐1.88)	1.57 (0.94‐2.63)
Atrial fibrillation	1.03 (0.74‐1.44)	0.86 (0.68‐1.07)	1.19 (0.90‐1.57)	1.01 (0.79‐1.28)	1.02 (0.88‐1.17)	1.16 (0.94‐1.42)	0.82 (0.47‐1.45)	1.17 (0.87‐1.56)	1.18 (0.78‐1.80)
All‐cause mortality	1.59 (1.23‐2.05)	1.49 (1.26‐1.77)	2.63 (2.19‐3.17)	1.52 (1.27‐1.82)	1.39 (1.23‐1.57)	1.97 (1.69‐2.29)	1.48 (1.11‐1.97)	1.06 (0.86‐1.30)	1.43 (1.08‐1.88)

*Note*: Model includes age, period of diagnosis, cancer stage, education, comorbidity index.

Abbreviations: CI, confidence interval; CVD, cardiovascular disease; HR, hazard ratio; PCa, prostate cancer.

### 3.4 Additional sensitivity analysis

We performed a sensitivity analysis by removing patients that were diagnosed in the year 2008, as some of the patients may not have complete CVD risk factor data in the 1‐year period before PCa diagnosis. There was very little change in the HRs and CIs. There was also no change in the results of models evaluating CVD risk when patients that received LHRH antagonists were removed (data not shown).

## 4 DISCUSSION

In our study of 30 923 patients with prostate cancer, 8449 received ADT as primary treatment within the first year of diagnosis. We identified an association between hormonal treatment and the composite CVD outcome, as well as the individual components myocardial infarction, stroke and heart failure. The association with the composite outcome was maintained in the low and moderate CVD risk groups. A stronger association was indicated in the high CVD risk group but was not statistically significant.

When we evaluated ADT based on duration (<7 months, 7‐18 months and 18+ months) the most prominent associations were in those with 18+ months ADT. When the analysis was stratified by ADT duration and CVD risk, there remained an association between ADT and composite CVD in the low‐risk and moderate‐risk groups, but only with the longest ADT duration. However, the stratified analyses often had lower numbers, so these analyses cannot clearly reject an association, due to lack of power.

Our interpretation of the lack of statistical significance in the high‐risk CVD group for the composite CVD outcome is mainly that there was a lack of power. There was a higher point estimate but wider confidence interval, and the number of patients in this group was significantly lower than the other two risk groups. Although Table 1 does indicate higher rates of CVD risk factors, and therefore treatment for these risk factors, we do not believe this was a strong factor in the high‐risk CVD group as there were high rates of both antihypertensive and antihypercholesterolemia medical treatment in this population that did not differ by ADT treatment status. To further evaluate possible confounding and bias in this analysis, we removed the small proportion of patients that received LHRH antagonists in post hoc sensitivity analysis to determine if this could have been a contributing factor, but there were no changes found in the interpretation of the results.

When interpreting the findings around duration of ADT treatment, we want to focus on the fact that the effect modification by treatment duration is likely because those receiving ADT for longer durations have a more severe form of PCa than those with shorter durations. Therefore, these findings could indicate the increased risk of CVD is reflective of an unhealthier population and not the treatment, despite controlling for comorbidity and cardiovascular risk factors.

We consistently identified an association between ADT and all‐cause mortality for all risk group and treatment durations, but caution interpretation of these findings as a direct cause of the treatment, due to the presence residual confounding of PCa severity not only being possible, but likely.

Our study benefits from nation‐wide registry data of high quality and contributes to a growing research field that seeks to identify subpopulations of people with PCa that may be at higher risk of CVD after treatment with ADT. Due to the nature of population‐wide data the findings have strong external generalisability. The study included 10 years of data for 30 923 PCa patients, with linkages between several health and sociodemographic registries to provide a rich amount of information. Data from the Cancer Registry of Norway, used to define the population, the Norwegian Prescription Database, used to create the treatment groups and risk factors, and the Norwegian Patient Registry, to identify outcomes, are of superior quality in terms of completeness and correctness.^18^, ^19^, ^20^, ^21^ This strength in data and large study size is reflected in the relatively narrow confidence intervals even when conducting stratified analysis of smaller groups.

One main concern was confounding by indication as PCa patients that have more advanced cancer and thus poorer overall survival, are more likely to receive ADT. These patients may also have a high burden of CVD risk factors, therefore complicating the ability to clearly determine an association.^22^, ^23^ Our main concern is for the mortality outcome and less for the cardiovascular disease outcomes as we used detailed diagnosis codes and medications to control for previous risk factors. Although we did not have access to cancer data specific to aggressiveness, other than stage, that would help to address this better, such as Gleason score or TNM staging, we attempted to address this bias concern by controlling for stage, comorbidity and specific CVD risk factors. In addition, treatment duration can be considered an indicator of disease severity as current guidelines have longer durations of hormonal treatment as severity increases.^24^

Despite efforts to control for confounding by indication, we believe there remained a lack of health status characterisation which has prohibited full understanding of the effect of the treatment, specifically for the all‐cause mortality outcome. This problem would be a concern for most studies evaluating this research question as even if one had increased data to better characterise the population, patients that receive ADT would inherently be an unhealthier population as it is a treatment recommended in more severe forms of PCa. Future studies should focus on a narrower population with advanced forms of the disease to create balanced exposure groups based on PCa diagnostic variables including Gleason score, TNM and stage, as well as cardiovascular risk factors and multimorbidity scoring. Propensity score matching may be an alternative design approach for observational studies evaluating, such as was done in a Korean study evaluating the impact of ADT on cerebral infarction.^25^

Additionally, we tried to reduce the risk of overt immortal time bias by requiring at least 1 year of follow‐up after diagnosis to account for waiting time before treatment started.^26^ While this method did allow sufficient time for patients to begin primary treatment there was an inevitable loss of outcome events within the first year after diagnosis (1 death and 1048 composite CVD events). Therefore, our analysis was evaluating longer term outcomes rather than those related to initial treatment onset. In addition, treatment duration was included as a time‐varying variable in the appropriate analyses.

Other possible concerns include relying on prescription data without evidence of adherence, not having data on smoking status, which is an important risk factor for CVD, but we did include education status and multimorbidity, which can capture some of the residual variation due to smoking. Also, there may be confounding by concomitant treatments, which we could not control for as we did not have data on other PCa treatments such as prostatectomy or radiotherapy. Rates of orchiectomy, as a surgical form of ADT, for primary treatment of PCa are very low in Norway, so we were not concerned with treatment bias.^27^ Finally, some of the stratified analyses had small numbers, which should be considered when interpreting the results.

A study from Sweden, based on national registry data, compared cardiovascular outcomes in people with PCa that received ADT to a PCa‐free population and found use of GnRH agonists to be associated with incident CVD events.^28^ This association was maintained in those without previous CVD and was stronger in those that had a history of CVD.

An observational study from Taiwan, which also aimed to evaluate the difference in survival benefit based on baseline CVD after ADT, found that ADT had an overall benefit on survival, but not for patients with prior CVD.^29^ Our study consisted of 3835 patients aged 65 and above with aggressive PCa, many with metastatic disease, which we excluded in our analyses.

Keating et al. evaluated the risk of myocardial infarction in a large, observational study of 185 106 people with local or regional PCa and found an association between ADT and the outcome, which was stronger in those with baseline CVD.^30^ However, they also found increased risk of myocardial infarction in those that did not receive ADT but had baseline CVD. The authors concluded there was no evidence that the excess risk of myocardial infarction in the ADT group was not modified by preexisting CVD and therefore treatment with ADT should not be based on preexisting risk, which disagrees with our own findings. Similarly, our study does not have congruent findings with two other studies that identified increased risk of all‐cause mortality in those with previous myocardial infarction or stroke^12^ or heart failure or myocardial infarction.^13^

A recent observational study from South Korea displayed findings contradictory to ours, but our study excluded patients with prior CVD, which constitutes a significant population.^10^ Also, their findings that ADT may reduce the risk of CVD in people that received ADT for more than 2 years demonstrate that in people with aggressive disease that require long‐term ADT, this type of treatment is necessary to prevent cancer specific mortality and not necessarily that ADT is cardio‐protective.

It remains not fully understood how ADT may directly cause serious cardiovascular events, but there is evidence that ADT has a detrimental metabolic impact, can decrease insulin sensitivity and lead to dyslipidemia.^16^, ^31^, ^32^ A recent prospective study from China on 60 patients with PCa demonstrated ADT to have a significant impact on BMI, waist to hip ratio, body fat percentage and HDL cholesterol and metabolic syndrome, all important risk factors for CVD.^33^ A post hoc analysis from trial data provided evidence to support a decrease in levels of several proteins associated with plaque instability in participants that received LHRH agonists, and therefore can possibly lead to increased risk of plaque rupture.^34^

The use of ADT in treatment for advanced and aggressive PCa serves a very important role with clear evidence from phase III randomised controlled trials that the use of ADT in combination with radiotherapy, has definitive superiority to radiotherapy alone for survival and disease progression.^35^, ^36^, ^37^ Although there is evidence of ADT being associated with increased risk of CVD and all‐cause mortality, it cannot simply be removed as a treatment option. Rather, risk reduction and mitigation strategies should be explored, specifically for subgroups of people at moderate risk of CVD.

Most importantly, there should be a focus on using clear guidelines to recommend ADT only to patients that have a demonstrated benefit from the treatment.^29^ An Italian cohort study demonstrated that roughly a quarter of their study population of people with PCa that were treated with ADT should not have been offered the treatment according to the European Association of Urology (EAU) guidelines.^38^ This group of patients treated with ADT discordant with EAU guidelines were also more at risk of CVD outcomes.

If a patient is at higher risk of CVD and requires ADT, then considering reducing duration of treatment may help mitigate risk.^39^, ^40^ In addition, risk mitigation could include careful monitoring and control of modifiable risk factors such as blood pressure, cholesterol and diabetes and emphasis on exercise and cognitive behaviour strategies that have been used to mitigate negative metabolic consequences of ADT.^23^, ^41^, ^42^, ^43^, ^44^

Since the introduction of newer LHRH antagonist medications as an option for ADT, research has begun exploring differences in cardiovascular risk with more traditional LHRH agonists with some emerging evidence of reduced risk of CVD with the newer antagonists.^34^, ^45^, ^46^, ^47^, ^48^ In one post hoc analysis, the cardiac biomarker N‐terminal pro‐B‐type natriuretic peptide (NTproBNP) at baseline was associated with increased risk of developing cardiovascular events in the LHRH agonist treatment arm but not the antagonist group.^49^ These findings need further investigation but could offer a biomarker for clinicians to identify patients more suitable for treatment with an LHRH antagonist. Results are currently awaited from the PRONOUNCE trial, a large randomised trial of 545 people evaluating major cardiovascular events between LHRH agonist vs antagonist treated patients.^50^

To summarise, in this unique study using registry‐based data, there is evidence of an association between ADT for nonmetastatic PCa and a higher risk of CVD events, especially in those with some CVD risk factors present at the time of their diagnosis and longer duration of treatment. We also found higher all‐cause mortality among patients receiving ADT. However, further studies with more detailed cancer data, specifically on cancer aggressiveness, are needed to determine the clinical relevance of these finding in context with overall benefit of ADT. Clinicians should carefully weigh the risks and benefits of ADT when developing a treatment plan, with possible consideration of early intervention for CVD risk factors.

## AUTHOR CONTRIBUTIONS

Rachel B. Forster contributed to the conceptualisation of the study and development of the methodology, in addition to project administration and writing the original draft as well as review and editing the draft for submission. Anders Engeland was vital in the conceptualization of the project, data curation, formal analysis, as well as reviewing and editing of the manuscript. Rune Kvåle contributed to the conceptualization of the study, funding acquisition and supervision, as well as input in to methodology and reviewing and editing of the manuscript. Vidar Hjellvik contributed to funding acquisition, data curation, formal analysis, input in to methodology and reviewing and editing of the manuscript. Tone Bjørge was vital in conceptualisation of the study, funding acquisition, supervision as well as review and editing of the manuscript. The work reported in the paper has been performed by the authors, unless clearly specified in the text.

## CONFLICT OF INTEREST

The authors declare no potential conflict of interests.

## ETHICS STATEMENT

The study was approved by the Regional Committee for Medical and Health Research Ethics (REC South East; no. 2010/131). Individual consent to use registry data for our study was not required under the Norwegian Health Research Act of 2008.

## Supporting information


**Appendix S1** Supporting Information.

## Data Availability

The data that support the findings of our study are available from the corresponding author upon reasonable request.
