# Supplementary material for: Association between medical androgen deprivation therapy and long‐term cardiovascular disease and all‐cause mortality in nonmetastatic prostate cancer
Source: Int J Cancer. 2022 May 17;151(7):1109–19. doi: 10.1002/ijc.34058 (PMC9544783; doi:10.1002/ijc.34058)
Supplement: Supplementary file 1 — Appendix S1 Supporting Information. [file IJC-151-1109-s001.pdf]

Association between medical androgen deprivation therapy and long-term cardiovascular disease and all-cause mortality in non-metastatic prostate cancer

Forster RB, Engeland A, Kvåle R, Hjellvik V, Bjørge T

Supplemental Information Table of Contents

Supplemental A. Outcome definitions: 2

Supplemental B. Prescription based comorbidity index 3

Supplemental C. Androgen deprivation treatments used in this study along with recommended duration for a single prescription used to determine total duration 4

Supplemental A. Outcome definitions:

The following outcomes were defined using International Classification of Diseases-Revision 10 (ICD10) and the International Classification of Primary Care-version 2 (ICPC2) codes from the Norwegian Prescription Database and the Norwegian Patient Registry.

- Myocardial infarction (ICPC2 = K75 or ICD10 = I21 or I22)
- Stroke (ICPC2 = K90 or ICD10 = I61, I63 or I64)
- Atrial fibrillation (ICPC2 = K78 or ICD10 = I48)
- Heart failure (ICPC2 = K77 or ICD10 = I11.0, I13.0, I13.2 or I50)

CVD risk factor definitions:

- Myocardial infarction, stroke, heart failure and atrial fibrillation were defined the same as the outcomes above. Diabetes was defined as ICD10 E10 or E11, ICPC2 T89 or T90 or a reimbursed ATC code within the A10 grouping
- Hypertension was defined by ICPC2 = K86 or K87 or ICD10 = I10, I11, I12, I13, I15 and/or ATC codes listed below:

C02AC05 (moksonidin)	C08CA03 (isradipine)	C09CA03 (valsartan)
C02CA04 (doxazosin)	C08CA05 (nifedipine)	C09CA04 (irbesartan)
C02DB02 (hydralazin)	C08CA13 (lercanidipine)	C09CA06 (candesartan)
C03AA03 (hydrokloratiazid)	C08DB01 (diltiazem)	C09CA07 (telmisartan)
C03AB01 (bendroflumetiazid)	C08DA01 (verapamil)	C09CA08 (olmesartan)
C03DA01 (spironolakton)	C09AA01 (captopril)	C09DA01 (hydrokloratiazid and losartan)
C03EA01 (thiazide and amiloride)	C09AA02 (enalapril)	C09DA02 (hydrokloratiazid and eprosartan
C07AA05 (propranolol)	C09AA03 (lisinopril)	C09DA03 (hydrokloratiazid and valsartan)
C07AB03 (atenolol)	C09AA04 (perindopril)	C09DA04 (hydrokloratiazid and irbesartan)
C07AB02 (metoprolol)	C09AA05 (ramipril)	C09DA06 (hydrokloratiazid and candesartan)
C07AB07 (bisoprolol)	C09BA02 (hydrokloratiazid and lisinopril)	C09DA07 (hydrokloratiazid and telmisartan)
C07AG01 (labetolol)	C09BA03 (hydrokloratiazid and enalapril)	C09DA08 (hydrokloratiazid and olmesartan)
C07AG02 (carvedilol)	C09BB02 (lercanidipine and enalapril)	C09DB01 (amlodipine and valsartan)
C08CA01 (amplodipine)	C09CA01 (Losartan)	C09DB02 (amlodipine and Olmesartan)
C08CA02 (felodipine)	C09CA02 (eprosartan)	

- Hypercholesterolemia was defined as ICD10 = E78.0-E78.5 and/or ATC codes listed below:

C10AA01 (simvastatin)	C10AX13 (evolocumab)
C10AA02 (lovastatin)	C10AX14 (alirocumab)
C10AA03 (pravastatin)	C10AC01 (colestyramine)
C10AA04 (fluvastatin)	C10AC04 (colesevalam)
C10AA05 (atorvastatin)	C10BA02 (ezetimib/simvastatin)
C10AA07 (rosuvastatin)	C10BA05 (ezetimib/atorvastatin)
C10AX09 (ezetimib)	C10BA0 (ezetimibe/rosuvastatin

Supplemental B. Prescription based comorbidity index

The methods of Häppölä et al. were followed as closely as possible, but we removed medications that were specific to prostate cancer, specific to female patients, or were not prescribed during the time period of the study[22].

Comorbidity-index:

a) Reimbursed prescription of the following drugs during the last four years prior to diagnosis (each ATC-code **increases** the index by one, except that "Platelet" counts as one):
   A10AB01, A10BA02, A10BB01, A10BB12, A12BA01, B01AA03, Platelet (B01AC04, B01AC05, B01AC22, B01AC16, B01AC24 or B01AC25), B01AC06, B01AC07, C01AA05, C03CA01, C03EA01, C07AB02, C07AB03, C07AB07, C07AG02, C08CA01, C08CA05, C09AA02, C09AA03, D07AC17, H05BA01, N02AX02, N04BA02, N05AA02, N05AD01, N05BA01, N05CF01, N06AB04, N06AX03, N06DA02, N06DX01, R03BB01, N05BA04, N05AB03, N05AX08, N06DA03, R03AC02, R03BB04, R03DA04
b) Reimbursed prescription of the following drugs during the last four years prior to diagnosis (each ATC-code **decreases** the index by one):
   C10AA01, C10AA05, C10AA07, N05BA06, N05CD08, N06AA09, N06AX11, R03AC03

After constructed, age-adjusted Cox regression was used to validate the association with all-cause mortality. As a numeric variable the index was associated with all-cause mortality (HR 1.3, 95% CI 1.3-1.4) and as a categorised variable, there was a clear increase in risk of death with the increasing index:

Index values	HR	95% CI
<0	0.9	0.8-1.0
0	1.0	Ref
1-4	1.8	1.7-2.0
5+	5.3	4.4-6.3

Supplemental C. Androgen deprivation treatments used in this study along with recommended duration for a single prescription used to determine total duration

Drug name, route, dosage	Frequency	Percent (%)	Recommended duration in one prescription
Eligard 2care4 inj subst 45mg	46	0.06	6 months
Eligard inj subst 7.5mg	49	0.07	1 month
Eligard inj subst 22.5mg	6069	8.17	3 months
Eligard inj subst 45mg	790	1.06	6 months
Enanton DepDu injsu 3.75mg sp	8	0.01	1 month
Enanton DepDu injsu 11.25mg sp	461	0.62	3 months
Enanton DepDu injsu 30mg spr	63	0.08	6 months
Enanton DepotSet injsu 3.75mg	8	0.01	1 month
Enanton DepotSet injsu 11.25mg	2751	3.70	3 months
Enanton DepotSet injsu 30mg	565	0.76	6 months
Firmagon inj subst 80mg	6354	8.55	1 month
Firmagon inj subst 120mg	627	0.84	1 month
Leuprorelin aur injsu 11.25mg	46	0.06	3 months
Leuprorelin san impl 5mg/spr	26	0.03	3 months
Procren Depot inj subs 3.75mg	26	0.03	1 month
Procren Depot inj subs 11.25mg	3026	4.07	3 months
Procren Depot inj subs 30mg	100	0.13	6 months
Vantas implantat 50mg	17	0.02	12 months - very rare
Zoladex abacus impl 3.6mg/spr	4	0.01	1 month
Zoladex farm implan 10.8mg/spr	5399	7.27	3 months
Zoladex implantat 3.6mg/spr	688	0.93	1 month
Zoladex implantat 10.8mg/spr	46048	61.98	3 months
Zoladex orif implan 10.8mg/spr	1044	1.41	3 months
Zoladex orif implantat 3.6mg	74	0.10	1 month
**Total**	74289		
